# Parental social contact in the work place and the risk of childhood acute lymphoblastic leukaemia

**DOI:** 10.1038/sj.bjc.6604024

**Published:** 2007-10-09

**Authors:** J S Chang, C Metayer, N T Fear, K Reinier, X Yin, K Urayama, C Russo, K W Jolly, P A Buffler

**Affiliations:** 1 Department of Epidemiology and Biostatistics, University of California, San Francisco, CA, USA; 2 Division of Epidemiology, School of Public Health, University of California, Berkeley, CA, USA; 3 King's College London, Academic Centre for Defence Mental Health, Institute of Psychiatry, London, UK; 4 Division of Cardiovascular Medicine, Oregon Health and Science University, Portland, OR, USA; 5 Department of Biostatistics, Boston University, Boston, MA, USA; 6 Department of Pediatrics, Kaiser Permanente Santa Clara Medical Center, Santa Clara, CA, USA; 7 Department of Pediatric Hematology/Oncology, Kaiser Permanente, Sacramento, CA, USA

**Keywords:** childhood leukaemia, parental occupation, social contact

## Abstract

To study the possible relation between parental social contact through occupation, a marker for a child's risk of infection, and childhood acute lymphoblastic leukaemia (ALL), the parents of 294 children with ALL aged 0–14.9 years and 376 matched controls were interviewed about their jobs after their child's birth up to the age of 3 years. Job titles were assigned to a level of social contact, and an index of occupational social contact months was created using the level and the job duration. Positive interactions between this index and rural residence associated with an increased risk of childhood ALL and common ALL (c-ALL) were observed (interaction *P*-value=0.02 for both, using tertiles of contact months; interaction *P*-value=0.05 and 0.02 for ALL and c-ALL, respectively, using continuous contact months); such findings were not observed when job durations were ignored. Our data suggest that duration of parental occupation may be important when examining the association between parental social contact in the workplace and childhood leukaemia.

Childhood leukaemia is the most common cancer among children in the United States, representing almost one-third of all cancer cases occurring in children under the age of 15 years (Ries *et al*, 1999). Although the causes of childhood leukaemia are still largely unknown, two major infectious hypotheses have been proposed, the ‘delayed infection’ (Greaves, 2006) and the ‘population mixing’ (Kinlen, 1995) hypotheses.

Studies have examined the association between childhood leukaemia and different proxy measures of child's risk of infection, including the daycare attendance (Ma *et al*, 2005) and birth order (Greaves, 2001). Seven childhood leukaemia studies have examined paternal occupational social contact as another proxy measure of child's risk of infection and the results have been inconsistent (Roman *et al*, 1994; Kinlen, 1997; Fear *et al*, 1999, 2005; Kinlen and Bramald, 2001; Kinlen *et al*, 2002; Pearce *et al*, 2004). The current analysis examines the association between childhood acute lymphoblastic leukaemia (ALL) and both paternal and maternal occupational social contact, incorporating the duration and the contact level of each job to determine whether adding the duration as part of the social contact measure will influence any association between parental social contact in the work place and ALL risk.

## MATERIALS AND METHODS

### Study population

The Northern California Childhood Leukaemia Study (NCCLS) is an ongoing case–control study that began in 1995. The study has been recruiting subjects from nine hospitals in the 35 counties located in the Northern California region, starting with 17 counties in the San Francisco Bay Area (years 1995–1999) and subsequently (since 1999) expanded to include 18 additional counties in the California Central Valley. For this analysis, NCCLS subjects included were recruited from 1995 to 2002 with leukaemia under the age of 15 and ascertained usually within 72 h of diagnosis. For each case, one or two controls matched on age, sex, Hispanic ethnicity, and maternal race were randomly selected based on birth certificates obtained through the California Office of Vital Records. The eligibility criteria for all subjects were (1) being a resident of the study area, (2) aged below 15 years at case diagnosis (reference date for the matched controls), (3) having at least one English- or Spanish-speaking parent or guardian, and (4) without previous cancer. Of the eligible cases, 84% consented to participate in the study and 84% of the eligible controls contacted agreed to participate in the study. Overall, 58% of the controls participated (the number of the enrolled controls divided by the total number of birth control searches excluding the known and presumed ineligibles). A detailed description of control selection has been published previously (Chang *et al*, 2006). The participating controls of the NCCLS are representative of the sampled population in parental age, parental education, and mother's reproductive history, which indicates a reduced potential for selection bias (Ma *et al*, 2004).

The study was approved by the University of California Committee for the Protection of Human Subjects, the California Health and Human Services Agency Committee for the Protection of Human Subjects, and the Institutional Review Boards of all the participating hospitals. A written informed consent was obtained from the parents of all participating subjects.

### Data collection and management

Data were collected from interviewed parents on employment history (and duration) of each job held after the child's birth up to the age of 3 years for those cases of children diagnosed from age 3 to 14.9 or up to the date of leukaemia diagnosis for those diagnosed under the age of 3 years. The diagnosed dates for the cases were used as the reference dates for exposure assessment for the matched control subjects. Each job was assigned a standard job title according to the 1990 US Census Occupational Classification (US Department of Health and Human Services, 1998). The level of occupational social contact was then assigned by the following two methods: first, the US job titles equivalent to the job titles of the UK 1990 Standard Occupational Classification were classified into three social contact groups (low, medium, and high) based on a previously published classification (Fear *et al*, 2005) (Supplementary Table 1). If occupations with different social contact levels were reported by the same person, the contact level was based on the job with the highest level. Those parents who did not work during the period of interest were assigned a low-level social contact. Like previous studies, this method does not include information on duration of employment. Second, an index was created by summarising each parent's employment history during the period of interest in the following equation: occupational social contact months=(months of employment with low social contact × 1)+(months of employment with medium social contact × 1.5)+(months of employment with high social contact × 2). In spite of the difference in social contacts between the three job contact categories, the factors for creating social contact months were chosen conservatively to give higher weightage to jobs with higher social contact levels. Sensitivity analyses were performed with different ranges of multiplication factors (see Supplementary Table 5).

A detailed method for classifying the rural/urban status has been published elsewhere (Reynolds *et al*, 2005). Briefly, each participant was assigned to a US Census block group according to birth residence and then assigned to one of the five urbanisation categories based on US Census 1990 and 2000 block group data for children born before 1 January 1995 and on or after 1 January 1995, respectively. For the purpose of this analysis, a dichotomous rural/urban status variable was created, which combined ‘urban’, ‘suburban’, ‘city’, and ‘small town’ into a more general urban group, while ‘rural’ included unpopulated areas and census-defined places with less than 50 000 people, outside of an urbanised area, with population density in the lowest quartile

### Statistical analysis

The present study included only the ALL cases diagnosed between ages 0 and 14.9. Analyses were performed first with all ALL cases and then separately with common ALL (c-ALL, which is defined as ALL with the expression of CD10 and CD19 surface antigens, and diagnosed in children aged 2–5.9 years; the 165 c-ALL cases in this study represent 92% of ALL cases at ages 2–5.9). Conditional logistic regression models were used to estimate the odds ratios.

For analyses with categorical predictor variables, analyses were first performed separately with paternal and maternal occupational social contact data using ‘low’ contact group as the reference with two indicator variables included in the statistical model for ‘medium’ and ‘high’ contact groups. Subsequent analyses were performed with a combined parental contact variable. The low exposure group in the combined parental analysis consisted of children whose both parents had a low contact level, the high exposure group consisted of children with either parent having a high contact job, and the remaining children were categorised as the medium group. For analyses with continuous predictor variables, the index of occupational social contact months was divided into tertiles with the lowest tertile as the reference group for comparison. Additional analyses were performed using contact months as a continuous variable estimating the odds ratio associated with one unit increment of social contact month.

Analyses examining the influence of rural/urban status on the association between parental contact and childhood ALL were performed using conditional logistic regression, first with paternal contact level for comparison with previous studies. Additional analyses were performed with combined parental occupational social contact. Subjects were categorised according to rural/urban status and the level of parental occupational social contact with those living in urban areas and having the lowest level of parental occupational social contact as the reference group. Product terms of rural/urban status and parental contact were included in the conditional logistic regression model. Log-likelihood ratio tests were used to compare the goodness-of-fit of the model with the product terms to the model without product terms.

All analyses were adjusted for household income, birth order, total child-hours spent in day care, and number of other children in the household before the index child attended the first grade. Since adjusting for parental educational level did not change risk estimates by more than 10%, these two variables were not included in the final statistical models. Although different associations were previously reported between daycare attendance and childhood leukaemia for Hispanic and non-Hispanic white children (Ma *et al*, 2005), statistical testing for interaction between Hispanic status and parental contact months indicated that the association with childhood ALL (*P*=0.81) or c-ALL (*P*=0.47) did not differ by Hispanic status; subjects of all race/ethnicity were therefore combined for these analyses.

Because of concern that positive results (Kinlen, 1997; Kinlen and Bramald, 2001; Kinlen *et al*, 2002) may reflect coding differences of contact level by job titles from those with null results (Fear *et al*, 1999, 2005), we repeated the same analyses using Kinlen's coding (Kinlen and Bramald, 2001) (Supplementary Table 2).

## RESULTS

The demographic characteristics of the study subjects are shown in Table 1. Controls had statistically significant (*P*<0.05) higher levels of household income and parental education.

Neither the paternal nor the maternal social contact through occupation was associated with either ALL or c-ALL for the analyses with the categorical exposure variable (low, medium, and high occupational social contact) or for the continuous variable (social contact month) (Table 2). Similarly, no significantly elevated risks were observed with either ALL or c-ALL with combined parental occupational social contact.

Table 3 presents the association between the paternal occupational social contact and ALL or c-ALL by rural/urban status. Analyses with categorical (low, medium, or high) paternal occupational social contact variable did not show a significant influence of rural/urban status on its association with childhood ALL or c-ALL (*P* for interaction=0.41 and 0.42, respectively). However, the analyses with social contact months showed a significant positive interaction between social contact months and living in rural areas for ALL (*P* for interaction=0.04 for tertiles and 0.09 for increment of one occupational social contact month) and for c-ALL (*P* for interaction=0.007 for tertiles and 0.02 for increment of one occupational social contact month). The same analysis with maternal data did not show any significant interaction between maternal occupational social contact and rural/urban status on the risk of ALL or c-ALL (data not shown).

An analysis examining the association between the combined parental occupational social contact and either ALL or c-ALL by rural/urban status showed similar results as those of paternal occupational social contact (Table 4). Analyses with categorical (low, medium, or high) parental occupational social contact variable did not show a significant influence of rural/urban status on its association with childhood ALL or c-ALL (*P* for interaction=0.55 and 0.86, respectively). However, the analyses with contact months showed a significant positive interaction between contact months and living in rural areas for ALL (*P* for interaction=0.02 for tertiles and 0.05 for increment of one occupational social contact month) and for c-ALL (*P* for interaction=0.02 for tertiles and 0.02 for increment of one occupational social contact month).

Tables 5 and 6 repeat the same analyses presented in Tables 2 and 4, respectively, but stratified by two age groups, ages 0–4.9 and 5–14.9. Though the point estimates were quite unstable due to smaller number of subjects after stratifying by age, the results in the two age groups were similar enough to justify combining the subjects from the two age groups for analysis.

The same analyses were repeated using coding by Kinlen and Bramald (2001) and the results were similar (Supplementary Tables 3 and 4). Sensitivity analyses were performed to test for the interaction between occupational social contact months and rural/urban status using three other combinations of multiplication factors for creating the index of occupational social contact months (1) 1.0 for both the medium and high social contact occupations; (2) 1.25 and 1.5 for medium and high social contact occupations, respectively; and (3) 2.0 and 3.0 for medium and high social contact occupations, respectively. Regardless of the multiplication factors used, the interaction between occupational social contact months and rural/urban status remained mostly statistically significant (*P*<0.05), especially for c-ALL (Supplementary Table 5). Interestingly, even when no weighting was assigned (multiplication factor 1.0 for both medium and high social contact occupation), there was still a significant interaction between rural/urban status and the duration of parental occupation on the risk of childhood leukaemia, which further support the importance of including duration as part of the parental occupational social contact measure.

## DISCUSSION

Seven published studies have examined parental social contact at work and the risk of childhood leukaemia, and the results have been inconsistent (Roman *et al*, 1994; Kinlen, 1997; Fear *et al*, 1999, 2005; Kinlen and Bramald, 2001; Kinlen *et al*, 2002; Pearce *et al*, 2004). The inconsistent results could be attributed to several sources. Most of those studies did not include information on maternal occupational contact level, since maternal occupation was not routinely recorded on available birth or death certificates (Kinlen, 1997; Fear *et al*, 1999, 2005; Kinlen and Bramald, 2001; Kinlen *et al*, 2002; Pearce *et al*, 2004). In addition, those studies only had information on father's employment at the following one or two points in time: at birth (Roman *et al*, 1994; Kinlen, 1997; Kinlen and Bramald, 2001; Kinlen *et al*, 2002; Pearce *et al*, 2004; Fear *et al*, 2005), at the child's death (Fear *et al*, 1999), or at diagnosis (Roman *et al*, 1994; Fear *et al*, 2005); none included information on job duration. This may be important in that the longer the duration the higher the probability of contact with carriers of infectious agents.

Our results suggest that duration of parental occupation may be important when examining the association between parental social contact in the workplace and childhood leukaemia. No statistically significant association between parental contact and either ALL or c-ALL was observed when job duration was not considered, which is consistent with three studies (Roman *et al*, 1994; Fear *et al*, 1999, 2005). In contrast, when duration of the occupation was accounted for, the results suggested that those children who live in a rural area have an increased risk of ALL or c-ALL associated with parents, specifically fathers, having occupations of higher social contact and longer duration; these results are consistent with four of the seven previously published studies (Kinlen, 1997; Kinlen and Bramald, 2001; Kinlen *et al*, 2002; Pearce *et al*, 2004) and support Kinlen's population mixing hypothesis (Kinlen, 1995). However, our own results (like those of Roman *et al*, 1994) need to be interpreted with caution because of the small number of subjects in rural areas, and the significant results were mainly attributed to father's exposure.

A major difference between our and Kinlen's studies conducted in Sweden and Scotland is that only 15% (39 cases and 45 controls) of our subjects lived in rural areas according to our definition, whereas Kinlen's studies had much larger numbers in rural areas (316 cases and 924 controls for the Scottish study; 196 cases aged 0–4 years and 784 controls for the Swedish study) (Kinlen and Bramald, 2001; Kinlen *et al*, 2002). In addition, the most rural counties in the Swedish study have a very low population density of 4 km^−2^ (Kinlen *et al*, 2002), whereas in our study, even the county with the lowest density (Glenn County) had a population density of 7.3 km^−2^ according to the 1990 US Census (US Census Bureau, 1990). Geographical isolation is also relevant to the population mixing hypothesis, and the rural counties in our study may be less geographically isolated than the rural areas in Sweden or Scotland, so that any association with parental occupational contact level would be correspondingly reduced.

Several issues are relevant in interpreting our results they are as follows: (1) a positive association between occupational social contact and childhood ALL or c-ALL in rural areas was observed with paternal but not with maternal exposure. The job contact levels were developed for paternal occupations and may have captured paternal exposures more accurately; however, it is unlikely that fathers and mothers with the same job titles (e.g., teachers and physicians) experience different levels of social contact. Another possible reason is that mothers, in general, have lower level of occupational social contact months (median=36.5 social contact months for control fathers *vs* median=27 for control mothers; 7.7% of control fathers *vs* 25.5% of mothers did not work during the period of interest). Finally, one can not rule out chance in the significant interaction between paternal contact months and rural/urban status due to the small number of rural subjects; however, the strength of interaction generally seems stronger for c-ALL, a subtype claimed as more associated with an infectious aetiology (Greaves, 2006), and thus decrease the probability that our results may be spurious. (2) All the fathers may not have lived with their child after birth, such details being available only for subjects recruited after 1999, among whom, 9% (34/378) of the fathers did not live in the same household as the child. The proportions of fathers who did not live in the same household as the child were similar between cases and controls (9.5% for cases and 8.7% for controls, *P* for *χ*^2^=0.80). Even so, they could still have had contact with their child, and thus, may have been an important source of exposure for childhood infections. Sensitivity analysis with subjects recruited after 1999, excluding those fathers who did not live in the same household as the child produced similar results. (3) Misclassification could also be a problem when assigning the level of social contact based on job titles, since not everyone with the same job title necessarily has the same level of social contact.

This study has several strengths, they are as follows: (1) it is one of the only two studies to investigate both paternal and maternal occupational contact level, which may involve less misclassification than using only paternal data; (2) it is the first study to take account of duration of occupation. Studies using parental occupation at a single point can misclassify the contact level, for example, a low contact job held for 12 months will be classified as a lower group than a high contact job for 1 month if based on birth certificate information, whereas by our index of social contact months, the reverse is the case. The concept is that with increasing duration of occupation, exposure to infection is more likely even if the job itself has a lower level of social contact. The index can also incorporate multiple jobs with different contact levels to produce a summary measure, thereby reducing the probability of misclassification. The duration information can accommodate breaks in employment, for example, taking time off work after the birth of their child; and (3) the cases appear to be representative of children in the 35-county study area. A previous comparison of the study with the California Cancer Registry indicated that 88% of childhood leukaemias were included in this study. NCCLS controls selected from birth certificates also appear to be representative of the population of the study area (Ma *et al*, 2004).

In conclusion, our results indicated that including information of the duration of parental occupation may be important when studying the association between parental occupational social contact and childhood leukaemia.

## Figures and Tables

**Table 1:** Characteristics of 294 ALL cases and 376 controls, the Northern California Childhood Leukaemia Study, 1995–2002

**Characteristics**	**Cases, *n* (%)**	**Controls, *n* (%)**	***P* †**
*Race/ethnicity* a			
Hispanic	124 (42.2)	157 (41.8)	—
Non-Hispanic white	135 (45.9)	169 (45.0)	
Others	35 (11.9)	50 (13.3)	
			
*Gender* a			
Male	152 (51.7)	192 (51.1)	—
Female	142 (48.3)	184 (48.9)	
			
*Age at diagnosis, years* a			
Mean (s.e.)	5.5 (0.2)	5.4 (0.2)	—
			
*Household income, $*			
<15 000	40 (13.6)	35 (9.3)	0.0009
15 000–29 999	59 (20.1)	54 (14.4)	
30 000–44 999	48 (16.3)	45 (12.0)	
45 000–59 999	52 (17.7)	58 (15.4)	
60 000–74 999	31 (10.5)	49 (13.0)	
⩾75 000	64 (21.8)	135 (35.9)	
			
*Maternal education*			
⩽High school	130 (44.2)	130 (34.6)	0.03
Some college	82 (27.9)	135 (35.9)	
⩾College	82 (27.9)	111 (29.5)	
			
*Paternal education*			
⩽High school	147 (51.0)	150 (41.3)	0.02
Some college	58 (20.1)	104 (28.7)	
⩾College	83 (28.8)	109 (30.0)	
			
*Maternal age at child's birth, years*			
Mean (s.e.)	28.0 (0.3)	28.8 (0.4)	0.10
			
*Paternal age at child's birth, years*			
Mean (s.e.)	30.8 (0.4)	31.1 (0.3)	0.59

ALL=acute lymphoblastic leukaemia.

a Matching variables.

† *P*-values were derived from *χ*^2^ tests for categorical variables and from Student's *t*-tests for continuous variables.

**Table 2:** Parental occupational social contact and risk of childhood ALL, the Northern California Childhood Leukaemia Study, 1995–2002

	**Total ALL**		**c-ALL**	
**Parental occupational contact level**	**Case/control**	**OR (95% CI)^a^**	**Case/control**	**OR (95% CI)^a^**
*Paternal*				
Low	194/223	Reference	106/122	Reference
Medium	41/66	0.95 (0.59–1.54)	24/39	1.18 (0.61–2.30)
High	59/87	0.92 (0.61–1.39)	35/55	0.90 (0.52–1.56)
				
*Occupational social contact months (tertiles)* b				
0–30.8	113/125	Reference	60/69	Reference
30.9–36.9	105/128	1.06 (0.70–1.60)	57/72	1.06 (0.60–1.88)
37.0 or more	76/123	0.95 (0.62–1.48)	48/75	1.16 (0.65–2.08)
Every 1 month increment		0.999 (0.990–1.007)		1.002 (0.991–1.013)
				
*Maternal*				
Low	169/214	Reference	98/121	Reference
Medium	25/31	1.56 (0.86–2.85)	13/14	1.77 (0.75–4.20)
High	99/129	1.14 (0.81–1.59)	53/79	1.03 (0.64–1.64)
				
*Occupational social contact months (tertiles)* b				
0–9.7	124/125	Reference	72/66	Reference
9.8–36.4	76/108	0.91 (0.60–1.39)	43/60	0.84 (0.48–1.46)
36.5 or more	93/141	0.92 (0.61–1.40)	49/88	0.70 (0.40–1.21)
Every 1 month increment		0.999 (0.992–1.006)		0.995 (0.985–1.004)
				
*Combined parental* c				
Low	113/135	Reference	64/69	Reference
Medium	45/62	1.34 (0.81–2.23)	28/36	1.62 (0.80–3.28)
High	135/177	1.21 (0.84–1.73)	72/109	1.10 (0.67–1.81)
				
*Occupational social contact months (tertiles)* b				
0–47.4	135/125	Reference	74/60	Reference
47.5–73.0	77/131	0.69 (0.46–1.04)	43/77	0.69 (0.41–1.16)
73.1 or more	81/118	1.05 (0.67–1.65)	47/77	0.87 (0.47–1.60)
Every 1 month increment		0.999 (0.993–1.004)		0.997 (0.990–1.005)

ALL=acute lymphoblastic leukaemia; c-ALL=common acute lymphoblastic leukaemia; CI=confidence interval; OR=odds ratio.

a The ORs were adjusted for annual household income, birth order, total child-hours spent in day care, and the number of other children in household before the index child attended first grade in school using conditional logistic regression.

b Occupational social contact months=(1.0 × months of employment with low social contact)+(1.5 × months of employment with medium social contact)+(2.0 × months of employment with high social contact).

c The ‘low’ exposure group in the combined parental analysis consists of children whose both parents had a low occupational contact level, the ‘high’ exposure group consists of children with either one of the parents having an occupation with high social contact level, and the remaining children are categorised as the ‘medium’ group.

**Table 3:** Paternal occupational social contact level and risk of childhood ALL by rural/urban status, the Northern California Childhood Leukaemia Study, 1995–2002

**Rural/urban**	**Paternal social contact^a^**	**Cases, *n* (%)**	**Controls, *n* (%)**	**OR (95% CI)^b^**	***P*-value for interactions**
*ALL*					
Urban	Low	138 (55.2)	154 (49.7)	Reference	
Urban	Medium	31 (12.4)	42 (13.5)	1.11 (0.62–1.98)	
Urban	High	42 (16.8)	69 (22.3)	0.85 (0.53–1.36)	
Rural	Low	25 (10.0)	30 (9.7)	0.93 (0.47–1.84)	
Rural	Medium	5 (2.0)	8 (2.6)	0.95 (0.24–3.73)	
Rural	High	9 (3.6)	7 (2.3)	1.82 (0.58–5.75)	0.41§
					
Urban	0–30.8^c^	85 (34.0)	83 (26.8)	Reference	
Urban	30.9–36.9	71 (28.4)	90 (29.0)	0.83 (0.51–1.35)	
Urban	37.0 or more	55 (22.0)	92 (29.7)	0.79 (0.47–1.33)	
Rural	0–30.8	12 (4.8)	20 (6.5)	0.50 (0.20–1.24)	
Rural	30.9–36.9	15 (6.0)	16 (5.2)	0.94 (0.39–2.26)	
Rural	37.0 or more	12 (4.8)	9 (2.9)	2.28 (0.76–6.85)	**0.04** §
Rural × occupational social contact months					0.09
					
					
*c-ALL*					
Urban	Low	76 (53.5)	87 (48.1)	Reference	
Urban	Medium	18 (12.7)	26 (14.4)	1.24 (0.56–2.70)	
Urban	High	24 (16.9)	41 (22.7)	0.83 (0.44–1.60)	
Rural	Low	13 (9.2)	17 (9.4)	0.88 (0.33–2.31)	
Rural	Medium	4 (2.8)	6 (3.3)	1.34 (0.26–7.05)	
Rural	High	7 (4.9)	4 (2.2)	2.23 (0.51–9.80)	0.42§
					
Urban	0–30.8^c^	46 (32.4)	43 (23.8)	Reference	
Urban	30.9–36.9	39 (27.5)	57 (31.5)	0.58 (0.29–1.17)	
Urban	37.0 or more	33 (23.2)	54 (29.8)	0.76 (0.37–1.56)	
Rural	0–30.8	6 (4.2)	13 (7.2)	0.25 (0.06–1.01)	
Rural	30.9–36.9	7 (4.9)	8 (4.4)	0.90 (0.26–3.18)	
Rural	37.0 or more	11 (7.8)	6 (3.3)	3.53 (0.86–14.57)	**0.007** §
Rural × occupational social contact months					**0.02**

ALL=acute lymphoblastic leukaemia; c-ALL=common acute lymphoblastic leukaemia; CI=confidence interval; OR=odds ratio.

a The ‘low’ exposure group in the combined parental analysis consists of children whose both parents had a low occupational contact level, the ‘high’ exposure group consists of children with either one of the parents having an occupation with high social contact level, and the remaining children are categorised as the ‘medium’ group.

b The ORs were adjusted for annual household income, birth order, total child-hours spent in day care, and the number of other children in household before the index child attended first grade in school using conditional logistic regression.

c Occupational social contact months=(1.0 × months of employment with low social contact)+(1.5 × months of employment with medium social contact)+(2.0 × months of employment with high social contact).

§ *P*-value was derived from log-likelihood ratio test comparing full model with interaction terms with the submodel without interaction terms. Bold values signifies *P*-values⩽0.05.

**Table 4:** Parental (paternal+maternal) occupational social contact level and risk of childhood ALL by rural/urban status, the Northern California Childhood Leukaemia Study, 1995–2002

**Rural/urban**	**Parental social contact^a^**	**Cases, *n* (%)**	**Controls, *n* (%)**	**OR (95% CI)^b^**	***P*-value for interactions**
*ALL*					
Urban	Low	83 (33.3)	91 (29.6)	Reference	
Urban	Medium	32 (12.9)	45 (14.6)	1.23 (0.66–2.28)	
Urban	High	96 (38.6)	128 (41.6)	1.12 (0.71–1.75)	
Rural	Low	14 (5.6)	21 (6.8)	0.80 (0.35–1.85)	
Rural	Medium	5 (2.0)	5 (1.6)	2.26 (0.54–9.43)	
Rural	High	19 (7.6)	18 (5.8)	1.38 (0.58–3.29)	0.55§
					
Urban	0–47.4^c^	99 (39.8)	82 (26.6)	Reference	
Urban	47.5–73.0	55 (22.1)	92 (29.9)	0.59 (0.36–0.96)	
Urban	73.1 or more	57 (22.9)	90 (29.2)	0.85 (0.49–1.46)	
Rural	0–47.4	16 (6.4)	23 (7.5)	0.51 (0.22–1.18)	
Rural	47.5–73.0	8 (3.2)	14 (4.6)	0.65 (0.23–1.84)	
Rural	73.1 or more	14 (5.6)	7 (2.3)	2.71 (0.86–8.56)	**0.02** §
Rural × occupational social contact months					**0.05**
					
					
*c-ALL*					
Urban	Low	44 (31.2)	51 (28.5)	Reference	
Urban	Medium	21 (14.9)	28 (15.6)	1.77 (0.76–4.12)	
Urban	High	53 (37.6)	74 (41.3)	1.43 (0.76–2.67)	
Rural	Low	8 (5.7)	10 (5.6)	1.36 (0.43–4.32)	
Rural	Medium	4 (2.8)	4 (2.2)	2.76 (0.52–14.63)	
Rural	High	11 (7.8)	12 (6.7)	1.38 (0.43–4.45)	0.86§
					
Urban	0–47.4^c^	52 (36.9)	39 (21.8)	Reference	
Urban	47.5–73.0	34 (24.1)	56 (31.3)	0.61 (0.33–1.13)	
Urban	73.1 or more	32 (22.7)	58 (32.4)	0.73 (0.34–1.53)	
Rural	0–47.4	8 (5.7)	13 (7.3)	0.40 (0.12–1.31)	
Rural	47.5–73.0	5 (3.6)	9 (5.0)	0.66 (0.17–2.61)	
Rural	73.1 or more	10 (7.1)	4 (2.2)	3.72 (0.69–20.16)	**0.02** §
Rural × occupational social contact months					**0.02**
					

ALL=acute lymphoblastic leukaemia; c-ALL=common acute lymphoblastic leukaemia; CI=confidence interval; OR=odds ratio.

a The ‘low’ exposure group in the combined parental analysis consists of children whose both parents had a low occupational contact level, the ‘high’ exposure group consists of children with either one of the parents having an occupation with high social contact level, and the remaining children are categorised as the ‘medium’ group.

b The ORs were adjusted for annual household income, birth order, total child-hours spent in day care, and the number of other children in household before the index child attended first grade in school using conditional logistic regression.

c Occupational social contact months=(1.0 × months of employment with low social contact)+(1.5 × months of employment with medium social contact)+(2.0 × months of employment with high social contact).

§ *P*-value was derived from log-likelihood ratio test comparing full model with interaction terms with the submodel without interaction terms. Bold values signifies *P*-values⩽0.05.

**Table 5:** Parental occupational social contact and risk of childhood ALL, the Northern California Childhood Leukaemia Study, 1995–2002, ages 0–4 and 5–14

	**Ages 0–4**		**Ages 5–14**	
**Parental occupational contact level**	**Case/control**	**OR (95% CI)^a^**	**Case/control**	**OR (95% CI)^a^**
*Paternal*				
Low	110/125	Reference	84/98	Reference
Medium	26/58	1.26 (0.66–2.40)	15/26	0.70 (0.32–1.53)
High	35/40	0.86 (0.51–1.46)	24/29	1.11 (0.57–2.18)
				
*Occupational social contact months (tertiles)* b				
0–30.8	73/81	Reference	40/44	Reference
30.9–36.9	53/67	0.84 (0.47–1.50)	52/61	1.37 (0.73–2.57)
37.0 or more	45/75	0.91 (0.51–1.61)	31/48	1.08 (0.53–2.20)
Every 1 month increment		0.996 (0.984–1.009)		1.002 (0.989–1.014)
				
*Maternal*				
Low	100/125	Reference	69/89	Reference
Medium	14/17	1.69 (0.75–3.80)	11/14	1.31 (0.53–3.24)
High	56/79	1.05 (0.66–1.67)	43/50	1.18 (0.71–1.97)
				
Occupational social contact months (tertiles)^b^				
0–9.7	77/71	Reference	47/54	Reference
9.8–36.4	46/80	0.66 (0.38–1.13)	30/28	1.65 (0.81–3.35)
36.5 or more	47/70	0.83 (0.46–1.52)	46/71	0.97 (0.53–1.78)
Every 1 month increment		0.997 (0.987–1.008)		0.999 (0.989–1.009)
				
*Combined parental* c				
Low	63/72	Reference	50/63	Reference
Medium	29/38	1.58 (0.81–3.08)	16/24	1.12 (0.49–2.54)
High	78/111	1.13 (0.71–1.82)	57/66	1.30 (0.74–2.29)
				
*Occupational social contact months (tertiles)* b				
0–47.4	82/71	Reference	53/54	Reference
47.5–73.0	47/84	0.65 (0.39–1.10)	30/47	0.77 (0.39–1.51)
73.1 or more	41/66	0.90 (0.48–1.68)	40/52	1.18 (0.60–2.31)
				
Every 1 month increment		0.997 (0.989–1.005)		1.000 (0.992–1.008)

ALL=acute lymphoblastic leukaemia; CI=confidence interval; OR=odds ratio.

a The ORs were adjusted for annual household income, birth order, total child-hours spent in day care, and the number of other children in household before the index child attended first grade in school using conditional logistic regression.

b Occupational social contact months=(1.0 × months of employment with low social contact)+(1.5 × months of employment with medium social contact)+(2.0 × months of employment with high social contact).

c The ‘low’ exposure group in the combined parental analysis consists of children whose both parents had a low occupational contact level, the ‘high’ exposure group consists of children with either one of the parents having an occupation with high social contact level, and the remaining children are categorised as the ‘medium’ group.

**Table 6:** Parental (paternal+maternal) occupational social contact level and risk of childhood ALL by rural/urban status, the Northern California Childhood Leukaemia Study, 1995–2002, ages 0–4 and ages 5–14

**Rural/urban**	**Parental social contact^a^**	**Cases, *n* (%)**	**Controls, *n* (%)**	**OR (95% CI)^b^**	***P*-value for interactions**
*Ages 0–4*					
Urban	Low	48 (31.8)	55 (28.7)	Reference	
Urban	Medium	22 (14.6)	28 (14.6)	1.54 (0.72–3.30)	
Urban	High	62 (41.1)	83 (43.2)	1.18 (0.68–2.07)	
Rural	Low	7 (4.6)	11 (5.7)	0.89 (0.28–2.81)	
Rural	Medium	3 (2.0)	5 (2.6)	1.15 (0.22–6.01)	
Rural	High	9 (6.0)	10 (5.2)	1.15 (0.36–3.68)	0.97§
					
Urban	0–47.4^c^	62 (41.1)	50 (26.0)	Reference	
Urban	47.5–73.0	39 (25.8)	64 (33.3)	0.59 (0.33–1.08)	
Urban	73.1 or more	31 (20.5)	52 (27.1)	0.72 (0.35–1.48)	
Rural	0–47.4	9 (6.0)	13 (6.8)	0.49 (0.16–1.55)	
Rural	47.5–73.0	3 (2.0)	11 (5.7)	0.26 (0.05–1.33)	
Rural	73.1 or more	7 (4.6)	2 (1.0)	2.81 (0.50–15.76)	**0.04** §
Rural × occupational social contact months					0.19
					
					
					
*Ages 5–14*					
Urban	Low	35 (35.7)	36 (31.0)	Reference	
Urban	Medium	10 (10.2)	17 (14.7)	0.70 (0.22–2.23)	
Urban	High	34 (34.7)	45 (38.8)	0.83 (0.38–1.84)	
Rural	Low	7 (7.1)	10 (8.6)	0.49 (0.13–1.82)	
Rural	Medium	2 (2.0)	0 (0.0)	—	
Rural	High	10 (10.2)	8 (6.9)	1.85 (0.44–7.81)	**0.03** §
					
Urban	0–47.4^c^	37 (37.8)	32 (27.6)	Reference	
Urban	47.5–73.0	16 (16.3)	28 (24.1)	0.54 (0.21–1.34)	
Urban	73.1 or more	26 (26.5)	38 (32.8)	1.04 (0.43–2.53)	
Rural	0–47.4	7 (7.1)	10 (8.6)	0.46 (0.12–1.73)	
Rural	47.5–73.0	5 (5.1)	3 (2.6)	2.19 (0.36–13.15)	
Rural	73.1 or more	7 (7.1)	5 (4.3)	2.70 (0.53–13.72)	0.06§
Rural × occupational social contact months					0.10
					

ALL=acute lymphoblastic leukaemia; CI=confidence interval; OR=odds ratio.

a The ‘low’ exposure group in the combined parental analysis consists of children whose both parents had a low occupational contact level, the ‘high’ exposure group consists of children with either one of the parents having an occupation with high social contact level, and the remaining children are categorised as the ‘medium’ group.

b The ORs were adjusted for annual household income, birth order, total child-hours spent in day care, and the number of other children in household before the index child attended first grade in school using conditional logistic regression.

c Occupational social contact months=(1.0 × months of employment with low social contact)+(1.5 × months of employment with medium social contact)+(2.0 × months of employment with high social contact).

§ *P*-value was derived from log-likelihood ratio test comparing full model with interaction terms with the submodel without interaction terms. Bold values signifies *P*-values⩽0.05.

## Supplementary Material

On-line Supplement Tables
