# Supplementary material for: Parental social contact in the work place and the risk of childhood acute lymphoblastic leukaemia
Source: Br J Cancer. 2007 Oct 9;97(9):1315–21. doi: 10.1038/sj.bjc.6604024 (PMC2360459; doi:10.1038/sj.bjc.6604024)
Supplement: On-line Supplement Tables [file 6604024x1.doc]

**On-line Supplement Tables**

On-line Supplement Table 1. Occupations according to 1990 US census occupational classification by level of occupational social contact using coding by Fear et al.*

| Level of occupational social contact | Occupational Title (1990 US census occupational classification code) |
| --- | --- |
| High | Managers, food service and lodging establishments (017); Managers, service organizations, not elsewhere classified (021); Physicians (084); Dentists (085); Veterinarians (086); Optometrists (087); Podiatrists (088); Registered nurses (095); Dieticians (97); Respiratory therapists (98); Occupational therapists (99); Physical therapists (103); Speech therapists (104); Therapists, not elsewhere classified (105); All postsecondary teachers (postsecondary = college or beyond) (113-119,123-129,133-139,143-149,153,154); Teachers, prekindergarten and kindergarten (155); Teachers, elementary school (156); Teachers, secondary school (157); Teachers, special education (158); Teachers, not elsewhere classified (159); Counselors, educational and vocational (163); Librarians (164); Social workers (174); Clergy (176); Religious workers, not elsewhere classified (177); Licenses practical nurses (207); Supervisors and proprietors, sales occupations  (243); Insurance sales occupations (253); Real estate sales occupations (254); Securities and financial services sales occupations (255); Advertising and related sales occupations (256); Sales occupations, other business services (257); Sales engineers (258); Sales representatives, mining, manufacturing and wholesale (259); Sales workers, motor vehicles and boats (263); Sales workers, apparel (264); Sales workers, shoes (265); Sales workers, furniture and home furnishings (266); Sales workers; hardware and building supplies (267); Sales workers, hardware and building supplies (268); Sales workers, parts (269); Sales workers, other commodities (274); Sales counter clerks (275); Cashiers (276); Street and door-to-door sales workers (277); News vendors (278); Demonstrators, promoters and models, sales (283); Auctioneers (284); Sales support occupations, not elsewhere classified (285); Hotel clerks (317); Transportation ticket and reservation agents (318); Receptionists (319); Library clerks (329); Postal clerks, exc. mail carriers (354); Mail clerks, exc. postal service (356); Messengers (357); Meter readers (366); Eligibility clerks, social welfare (377); Bill and account collectors (378); Bank tellers (383); Teachers' aids (387); Child care workers, private household (406); Supervisors, guards (415); Police and detectives, pubic service (418); Sheriffs, bailiffs, and other law enforcement officers (423); Correctional institution officers (424); Crossing guards (425); Protective services occupations, not elsewhere classified (427); Bartenders (434); Waiters and waitresses (435); Waiters'/waitresses' assistants (443); Dental assistants (445); Health aids, except nursing (446); Nursing aides, orderlies, and attendants (447); Elevator operators (454); Pest control occupations (455); Supervisors, personal service occupations (456); Barbers (457); Hairdressers and cosmetologists (458); Attendants, amusement and recreation facilities (459); Guides (461); Ushers (462); Public transportation attendants (463); Baggage porters and bellhops (464); Welfare service aids (465); Family child care providers (466); Early childhood teachers' assistants (467); Child care workers, not elsewhere classified (468); Personal service occupations, not elsewhere classified (469); Optical goods workers (677); Butchers and meat cutters (686); Driver-sales workers (806); Bus drivers (808); Taxicab drivers and chauffeurs (809); Parking lot attendants (813); Railroad conductors and yardmasters (823); Supervisors, handlers, equipment cleaners, and laborers, not elsewhere classified (864); Garage and service station representatives (885); |
|  |  |
| Medium | Legislators (003); Administrators and officials, public administration (005); Administrators, protective services (006); Managers, medicine and health (015);  Postmasters and mail superintendents (016); Managers, property and real estate (018); Managers and administrators, not elsewhere classified (022); Business and promotion agents (034); Management related occupations (037); Health diagnosing practitioners, not elsewhere classified (89); Pharmacists (096); Physicians' assistants (106); Psychologists (167); Recreation workers (175); Lawyers (178); Judges (179); Musicians and composers (186); Actors and directors (187); Photographers (189); Dancers (193); Artists, performers, and related workers, not elsewhere classified (194); Announcers (198); Athletes (199); Clinical lab technologists and technicians (203); Dental hygienists (204); Radiologic technicians (206); Health technologists and technicians, not elsewhere classified (208); Broadcast equipment operators (228); Legal assistants (234); Supervisors, general office (303); Launderers and ironers (403); Supervisors, police and detectives (414); Food counter and related occupations (438); Miscellaneous food preparation (444); Supervisors, mechanics and repairers (503); Automobile mechanics (505); Automobile mechanic apprentices (506); Bus, truck, and stationary engine mechanics (507); Aircraft engine mechanics (508); Small engine repairers (509); Aircraft mechanics, exc. engine (515); Heavy equipment mechanics (516); Farm equipment mechanics (517); Industrial machinery repairers (518); Machinery maintenance occupations (519); Electronic repairers, communications and industrial equipment (523); Household appliance and power tool repairers (526); Miscellaneous electrical and electronic equipment repairers (533); Specified mechanics and repairers, not elsewhere classified (547); Not specified mechanics and repairers (549); Electricians (575); Electrician apprentices (576); Dressmakers (666); Tailors (667); Shoe repairers (669); Shoe machine operators (745); Pressing machine operators (747); Laundering and dry cleaning machine operators (748); Motion picture projectionists (773); Supervisors, motor vehicle operators (803); Helpers, mechanics and repairers (865); |
|  |  |
| Low | All occupations not in the high or medium category |

* Reference: Fear NT, Simpson J, Roman E. Childhood cancer and social contact: the role of paternal occupation. Cancer Causes and Control 2005;16:1091-1097.

On-line Supplement Table 2. Occupations according to 1990 US census occupational classification by level of occupational social contact using coding by Kinlen et al.*

| Level of occupational social contact | Occupational Title (1990 US census occupational classification code) |
| --- | --- |
| Very High | Construction inspectors (35); Surveyor and mapping scientists (63); All postsecondary teachers (postsecondary = college or beyond) (113-119,123-129,133-139,143-149,153,154); Teachers, prekindergarten and kindergarten (155); Teachers, elementary school (156); Teachers, secondary school (157); Teachers, special education (158); Teachers, not elsewhere classified (159); Airplane pilots and navigators (226); Mail carriers, postal service (355); Teachers’ aides (387); Nursing aides, orderlies, and attendants (447); Family child care providers (466); Early childhood teacher’s assistants (467); Childcare workers (468); Heating, air conditioning, and refrigeration mechanics (534); Supervisors, carpenters and related workers (554); Supervisors, plumbers, pipefitters and steamfitters (557); Construction supervisors (558); Carpenters (567); Carpenter apprentices (569); Drywall installers (573); Plasterers (584); plumbers, pipefitters and steamfitters (585); plumbers, pipefitters and steamfitters apprentices (587); Glaziers (589); Insulation workers (593); Paving, surfacing, and tamping equipment operators (594); Roofers (595); Bridge, lock, and Drillers, earth (598); Construction trade, not elsewhere classified (599); Supervisors, Motor vehicle operators (803); Truck drivers (804); Bus drivers (808); Taxicab drivers and chauffeurs (809); Motor transportation occupations (814); Railroad conductors and yardmasters (823); Locomotive operating occupations (824); Rail vehicle operators (826); Ship captains and mates, except fishing boats (828); Marine engineers (833); lighthouse tenders (834); Supervisors, material moving equipment operators (843); Operating engineers (844); Longshore equipment operators (845); Hoist and winch operators (848); Crane and tower operators (849); Excavating and loading machine operators (853); Grader, dozer, and scraper operators (855); Miscellaneous material moving equipment operators (859); Construction laborers (869); |
|  |  |
| High | Administrators, protective services (6); Personnel and labor relations managers (8); Managers, marketing, advertising, and public relations (13); Administrators, education and related fields (14); Managers, medicine and health (15); Managers, food serving and lodging establishments (17); Funeral directors (19); Managers, service organizations, not elsewhere classified (21); Managers and administrators, not elsewhere classified (22); Business and promotion agents (34); Management and related occupations, not elsewhere classified (37); Physicians (84); Dentists (85); Veterinarians (86); Optometrists (87); Podiatrists (88); Health diagnosing practitioners, not elsewhere classified (89); Registered nurses (95); Pharmacists (96); Dietician (97); Respiratory therapist (98); Occupational therapist (99); Physical therapists (103); Speech therapist (104); Therapist, not elsewhere classified (105); Physicians’ assistants (106); Counselors, educational and vocational (163); Librarian (164); Psychologists (167); Social workers (174); Recreation workers (175); Clergy (176); Religious workers not elsewhere classified (177); Lawyers (178); Judges (179); Musicians and composers (186); Actors and directors (187); Photographers (189); Dancers (193); Artists, performers, and related workers, not elsewhere classified (194); Announcers (198); Athletes (199); Clinical laboratory technologists and technicians (203); Dental hygienists (204); Radiologic technicians (206); Licensed practical nurses (207); Health technologists and technicians (208); Broadcast equipment operators (228); Supervisors and proprietors, sales occupations (243); Insurance sales occupations (253); Real estate sales occupations (254); Securities and financial services sales occupations (255); Advertising and related sales occupations (256); Sales occupations, other business services (257); Sales workers, motor vehicles and boats (263); Sales workers, apparel (264); Sales workers, shoes (265); Sales workers, furniture and home furnishings (266); Sales workers, radio, TV, hi-fi, and appliances (267); Sales workers, hardware and building supplies (268); Sales workers, parts (269); Sales workers, other commodities (274); Sales counter clerk (275); Cashiers (276); Street and door-to-door sales workers (277); News vendors (278); Demonstrators, promoters and models, sales (283); Auctioneers (284); Sales support occupations, not elsewhere classified (285); Supervisors, general office (303); Eligibility clerks, social welfare (377); Child care workers, private household (406); Supervisors, firefighting and fire prevention occupations (413); Supervisors, police and detectives (414); Supervisors, guard (415); Fire inspection and fire prevention occupations (416); Firefighting occupations (417); Police and detectives, public service (418); Sheriffs, bailiffs, and other law enforcement officers (423); Correctional institution officers (424); Bartenders (434); Waiters and waitresses (435); Food counter, fountain, and related occupations (438); Waiter’/waitresses’ assistants (443); Miscellaneous food preparation occupations (444); Dental assistants (445); Elevator operators (454); Pest control occupations (455); Supervisors, personnel occupations (456); Barbers (457); Hairdressers and cosmetologists (458); Attendants, amusement and recreation facilities (459); Guides (461); Ushers (462); Public transportation attendants (463); Welfare service aides (465); Personal service occupations, not elsewhere classified (469); Telephone line installers and repairers (527); Supervisors, painters, paperhangers, and plasterers (556); Painters, construction and maintenance (579); Paperhangers (583); Dressmakers (666); Tailors (667); Shoe repairers (669); Optical goods workers (677); Butchers and meat cutters (686); Shoe machine operators (745); Painting and paint spraying machine operators (759); Motion picture projectionists (773); Hand painting, coating, and decorating occupations (789); Driver-sales worker (806); Parking lot attendants (813); Supervisors, handlers, equipment cleaners, and laborers, not elsewhere classified (864); Garage and service station related occupations (885); Commissioned officers and warrant officers (903); Non-commissioned officers and other enlisted personnel (904); Military occupation, rank not specified (905); |
|  |  |
| Medium or Low | All occupations not in the very high or high category |

* Reference: Kinlen LJ, S Bramald. Paternal occupational contact level and childhood leukaemia in rural Scotland: a

case-control study. British Journal of Cancer 2001;84:1002-1007.

On-line Supplement Table 3. Parental occupational social contact level and the risk of childhood ALL, the Northern California Childhood Leukaemia Study, 1995 to 2002, reanalysis based on Kinlen’s coding

|  |  | Total ALL | |  |  | c-ALL | |  |
| --- | --- | --- | --- | --- | --- | --- | --- | --- |
| Parental occupational contact level | Case/control | | OR (95% CI)* | | Case/control | | OR (95% CI)* | |
| **Paternal** |  | |  | |  | |  | |
| Low + Medium | 169/195 | | Reference | | 101/117 | | Reference | |
| High | 75/128 | | 0.86 (0.59-1.26) | | 36/71 | | 0.81 (0.48-1.36) | |
| Very High | 50/53 | | 1.19 (0.75-1.89) | | 28/28 | | 1.36 (0.73-2.55) | |
|  |  | |  | |  | |  | |
| Occupational social contact months (tertiles)† |  | |  | |  | |  | |
| 0 to 30.9 | 110/125 | | Reference | | 60/71 | | Reference | |
| 31 to 40.9 | 91/125 | | 1.00 (0.66-1.53) | | 52/69 | | 1.14 (0.64-2.05) | |
| 41 or more | 93/126 | | 1.17 (0.76-1.80) | | 53/76 | | 1.19 (0.69-2.04) | |
| Every one month increment |  | | 1.001 (0.993-1.010) | |  | | 1.005 (0.994-1.016) | |
|  |  | |  | |  | |  | |
| **Maternal** |  | |  | |  | |  | |
| Low + Medium | 172/225 | | Reference | | 101/127 | | Reference | |
| High | 93/113 | | 1.41 (0.99-2.02) | | 45/66 | | 1.18 (0.71-1.94) | |
| Very High | 28/36 | | 1.17 (0.66-2.08) | | 18/21 | | 1.33 (0.60-2.91) | |
|  |  | |  | |  | |  | |
| Occupational social contact months (tertiles)† |  | |  | |  | |  | |
| 0 to 9.0 | 124/125 | | Reference | | 72/66 | | Reference | |
| 9.1 to 36.4 | 82/122 | | 0.87 (0.58-1.32) | | 48/69 | | 0.82 (0.48-1.42) | |
| 36.5 or more | 87/127 | | 1.00 (0.65-1.52) | | 44/79 | | 0.72 (0.40-1.29) | |
| Every one month increment |  | | 0.999 (0.991-1.008) | |  | | 0.993 (0.981-1.005) | |
|  |  | |  | |  | |  | |
| **Combined parental‡** |  | |  | |  | |  | |
| Low + Medium | 102/129 | | Reference | | 62/74 | | Reference | |
| High | 117/162 | | 1.34 (0.92-1.96) | | 59/95 | | 1.20 (0.72-2.00) | |
| Very High | 74/83 | | 1.39 (0.90-2.15) | | 43/45 | | 1.47 (0.83-2.63) | |
|  |  | |  | |  | |  | |
| Occupational social contact months (tertiles)† |  | |  | |  | |  | |
| 0 to 45.4 | 120/124 | | Reference | | 68/60 | | Reference | |
| 45.5 to 76.9 | 77/112 | | 0.99 (0.66-1.49) | | 44/70 | | 0.85 (0.50-1.44) | |
| 77.0 or more | 96/138 | | 1.12 (0.73-1.72) | | 52/84 | | 0.89 (0.50-1.59) | |
| Every one month increment |  | | 1.000 (0.994-1.006) | |  | | 0.999 (0.991-1.007) | |

* The odds ratios from analyses before birth are adjusted for annual household income using conditional logistic regression. The odds ratios from analyses after birth are adjusted for annual household income, birth order, total child-hours spent in daycare, and the number of other children in household before the index child attended first grade in school using conditional logistic regression

† Occupational social contact months = (1.0 x months of employment with low social contact) + (1.5 x months of employment with medium social contact) + (2.0 x months of employment with high social contact)

**‡** The “low + medium” exposure group in the combined parental analysis consists of children where both parents had a low or medium occupational contact level, the “very high” exposure group consists of children with either one of the parents having an occupation with a very high social contact level, and the remaining rest children are categorized as the “medium” group

On-line Supplement Table 4. Parental (Paternal + Maternal) occupational social contact level by rural/urban status and the risk of childhood ALL, the Northern California Childhood Leukaemia Study, 1995 to 2002, reanalysis based on Kinlen’s coding

| **Rural /Urban** | **Social contact*** | **Cases**  **n (%)** | **Controls**  **n (%)** | **OR adjusted†** | ***p-value* for interactions** |
| --- | --- | --- | --- | --- | --- |
| **ALL** | |  |  |  |  |
| Urban | Low + medium | 74 (29.7) | 87 (28.3) | Reference |  |
| Urban | High | 85 (34.1) | 118 (38.3) | 1.26 (0.80-1.98) |  |
| Urban | Very high | 52 (20.9) | 59 (19.2) | 1.34 (0.78-2.28) |  |
| Rural | Low + medium | 11 (4.4) | 21 (6.8) | 0.66 (0.26-1.65) |  |
| Rural | High | 13 (5.2) | 15 (4.9) | 1.73 (0.70-4.30) |  |
| Rural | Very high | 14 (5.6) | 8 (2.6) | 2.57 (0.85-7.75) | *p =* 0.28 § |
|  |  |  |  |  |  |
| Urban | 0 to 45.4 **‡** | 88 (35.3) | 81 (26.3) | Reference |  |
| Urban | 45.5 to 76.9 | 51 (20.5) | 79 (25.7) | 0.78 (0.48-1.26) |  |
| Urban | 77.0 or more | 72 (28.9) | 104 (33.8) | 1.04 (0.62-1.73) |  |
| Rural | 0 to 45.4 | 13 (5.2) | 21 (6.8) | 0.53 (0.23-1.24) |  |
| Rural | 45.5 to 76.9 | 11 (4.4) | 13 (4.2) | 1.25 (0.45-3.42) |  |
| Rural | 77.0 or more | 14 (5.6) | 10 (3.3) | 2.10 (0.73-6.08) | *p =* 0.06 § |
|  | |  |  |  |  |
| Rural x occupational social contact months | |  |  |  | *p* = 0.07 |
|  | |  |  |  |  |
| **c-ALL** | |  |  |  |  |
| Urban | Low + medium | 43 (30.5) | 54 (30.2) | Reference |  |
| Urban | High | 46 (32.6) | 68 (38.0) | 1.41 (0.76-2.60) |  |
| Urban | Very high | 29 (20.6) | 31 (17.3) | 1.80 (0.86-3.76) |  |
| Rural | Low + medium | 6 (4.3) | 11 (6.2) | 0.88 (0.24-3.22) |  |
| Rural | High | 7 (5.0) | 9 (5.0) | 1.52 (0.46-4.99) |  |
| Rural | Very high | 10 (7.1) | 6 (3.4) | 2.77 (0.73-10.56) | *p =* 0.82 § |
|  |  |  |  |  |  |
| Urban | 0 to 45.4 **‡** | 49 (34.8) | 39 (21.8) | Reference |  |
| Urban | 45.5 to 76.9 | 30 (21.3) | 47 (26.3) | 0.68 (0.36-1.27) |  |
| Urban | 77.0 or more | 39 (27.7) | 67 (37.4) | 0.82 (0.42-1.62) |  |
| Rural | 0 to 45.4 | 6 (4.3) | 11 (6.2) | 0.33 (0.09-1.25) |  |
| Rural | 45.5 to 76.9 | 8 (5.7) | 11 (6.2) | 1.04 (0.31-3.46) |  |
| Rural | 77.0 or more | 9 (6.4) | 4 (2.3) | 3.62 (0.64-20.52) | *p =* **0.02** § |
|  |  |  |  |  |  |
| Rural x occupational social contact months | |  |  |  | *p* = **0.03** |

* The “low + medium” exposure group in the combined parental analysis consists of children where both parents had a low or medium occupational contact level, the “very high” exposure group consists of children with either one of the parents having an occupation with a very high social contact level, and the remaining rest children are categorized as the “medium” group

**†** The Odds ratios from analyses before birth are adjusted for annual household income using conditional logistic regression. The Odds ratios from analyses after birth are adjusted for annual household income, birth order, total child-hours spent in daycare, and the number of other children in household before the index child attended first grade in school using conditional logistic regression

**‡** Occupational social contact months = (1.0 x months of employment with low social contact) + (1.5 x months of employment with medium social contact) + (2.0 x months of employment with high social contact)

§ p value was derived from log-likelihood ratio test comparing full model with interaction terms with the submodel without interaction terms.

On-line Supplement Table 5. P values for testing the interaction between rural/urban status and parental social contact through occupation using different combination of multiplication factors to create the index of occupational social contact months

|  | Index 1 | Index 2 | Index 3 | Index 4 |
| --- | --- | --- | --- | --- |
| Rural x duration | Med x 1.0; High x 1.0 | Med x 1.25; High x 1.5 | Med x 1.5; High x 2.0 | Med x 2.0; High x 3.0 |
| **Fear’s coding*** |  |  |  |  |
| **ALL** |  |  |  |  |
| Analysis with tertiles | *p* = **0.02** | *p* = **0.02** | *p* = **0.02** | *p* = **0.04** |
|  |  |  |  |  |
| Analysis with continuous variable (months) | *p* = 0.09 | *p* = 0.06 | *p* = **0.05** | *p* = **0.05** |
|  |  |  |  |  |
| **c-ALL** |  |  |  |  |
| Analysis with tertiles | *p* = **0.03** | *p* = **0.04** | *p* = **0.02** | *p* = **0.02** |
|  |  |  |  |  |
| Analysis with continuous variable (months) | *p* = **0.03** | *p* = **0.02** | *p* = **0.02** | *p* = **0.03** |
|  |  |  |  |  |
| **Kinlen’s coding†** |  |  |  |  |
| **ALL** |  |  |  |  |
| Analysis with tertiles | *p* = 0.06 | *p* = **0.003** | *p* = 0.06 | *p* = **0.02** |
|  |  |  |  |  |
| Analysis with continuous variable (months) | *p* = 0.12 | *p* = 0.07 | *p* = 0.07 | *p* = 0.07 |
|  |  |  |  |  |
| **c-ALL** |  |  |  |  |
| Analysis with tertiles | *p* = 0.09 | *p* = **0.008** | *p* = **0.02** | *p* = **0.01** |
|  |  |  |  |  |
| Analysis with continuous variable (months) | *p* = **0.04** | ***p* = 0.03** | *p* = **0.03** | *p* = **0.04** |

* Reference: Fear NT, Simpson J, Roman E. Childhood cancer and social contact: the role of paternal occupation. Cancer Causes and Control 2005;16:1091-1097.

† Reference: Kinlen LJ, S Bramald. Paternal occupational contact level and childhood leukaemia in rural Scotland: a

case-control study. British Journal of Cancer 2001;84:1002-1007.
